# Targeting of oxidized Macrophage Migration Inhibitory Factor (oxMIF) with antibody ON104 attenuates the severity of glomerulonephritis

**DOI:** 10.1371/journal.pone.0311837

**Published:** 2024-10-07

**Authors:** Maroua Ferhat, Julia Mayer, Lyndon H. Costa, Maria Prendecki, Alejandro A. Puchol Tarazona, Alexander Schinagl, Randolf J. Kerschbaumer, Frederick W. K. Tam, Christine Landlinger, Michael Thiele

**Affiliations:** 1 OncoOne Research & Development GmbH, Vienna, Austria; 2 Department of Immunology and Inflammation, Centre for Inflammatory Disease, Hammersmith Hospital, Imperial College London (ICL), London, United Kingdom; University of KwaZulu-Natal, SOUTH AFRICA

## Abstract

The oxidized form of Macrophage Migration Inhibitory Factor (oxMIF) has been identified as the disease-related isoform of MIF, exerting pathological functions in inflamed tissue. In this study, we aimed to explore the *in vivo* effects of the neutralizing anti-oxMIF antibody ON104 in a rat model of crescentic glomerulonephritis (CGN), to better understand its disease modifying activities. WKY rats received a single intravenous injection of a rabbit nephrotoxic serum (NTS), targeting rat glomerular basement membrane to induce CGN. On day 4 and day 6, ON104 was given intraperitoneally (*i*.*p*.) and on day 8 urine, blood and kidney tissue were collected. ON104 substantially attenuated the severity of CGN demonstrated by reduced proteinuria, hematuria, as well as lower levels of kidney injury molecule (KIM)-1. ON104 treatment preserved the glomerular morphology and suppressed crescent formation, a hallmark of the disease. On the cellular level, oxMIF neutralization by ON104 strongly reduced the number of macrophages and neutrophils within the inflamed kidneys. *In vitro*, we identified human neutrophils, but not monocytes, as main producers of oxMIF among total peripheral cells. The present study demonstrates that oxMIF is a pertinent therapeutic target in a model of CGN which mechanistically resembles human immune mediated CGN. In this model, neutralization of oxMIF by ON104 leads to an improvement in both urinary abnormalities and histological pathological characteristics of the disease. ON104, thus has the potential to become a novel disease-modifying drug for the treatment of glomerulonephritis and other inflammatory kidney diseases.

## Introduction

Glomerulonephritis (GN) covers a heterogeneous group of immune-mediated kidney disorders characterized by inflammation and structural alterations mainly within the glomeruli. Crescentic glomerulonephritis (CGN) is the most severe manifestation of GN, characterized by extensive glomerular crescents (in more than 50% of the glomeruli) which affects people of all ages and ethnicities worldwide [1–3]. Despite significant advances in understanding the disease pathophysiology and in therapeutic approaches, GN remains an area of high unmet medical need, as it continues to be associated with significant morbidity and mortality, including end-stage renal disease (ESRD) and cardiovascular complications [4,5]. The prognosis of GN varies depending on the underlying disease and the baseline comorbidity of the patient.

The intricate interplay between immune cell populations and molecular mediators is central to the pathogenesis of GN, progression and severity. One such pivotal player is Macrophage Migration Inhibitory Factor (MIF), described as a proinflammatory cytokine with versatile roles in immune responses and tissue homeostasis [6–9]. Transcriptional regulation of MIF is influenced by genetic factors, and its increased expression is linked to autoimmune diseases and inflammatory responses [7,10]. MIF is produced by various kidney cells and has been associated with both inflammatory and constitutive functions in kidney injury [7,10,11]. In healthy human kidneys, MIF is expressed by proximal renal tubular epithelial cells, parietal epithelial cells (PECs), mesangial cells and in Bowman’s capsular epithelial cells or podocytes [12,13]. During progressive GN, renal expression of MIF, and its receptor CD74, is dysregulated [13,14]. Elevation of urine and serum MIF levels in patients with proliferative GN, systemic lupus erythematosus (SLE) or membranous nephropathy (MN) correlate with disease severity and are associated with more severe pathological features [15–20]. Two promoter polymorphisms in *MIF* genes: -173G/C (rs755622) and -794 CATT(5–8) microsatellite repeat (rs5844572) have been linked to susceptibility to SLE [21–23]. In rodents, MIF or CD74 genetic deficiency confers protection against CGN [13,24]. Moreover, indirect inhibition of MIF using endogenous molecules, such as Ribosomal Protein 19 (RSP19), was shown to suppress MIF’s interaction with CD74, to decrease MIF levels, to prevent monocyte adherence to endothelial cells, and to restrict ERK and AKT phosphorylation, *in vitro* [25]. Similarly, miRNAs (miR-654) downregulated MIF and cytokine expression, reduced ERK and AKT phosphorylation, and limited cell proliferation [18]. *In vivo*, activation of miR-654 or treatment with recombinant RSP19 suppresses immune cell infiltration, cytokine production, and downstream signaling which protected mice against CGN-induced kidney injury [18,25].

Despite its clear role in GN pathogenesis, MIF represents a challenging therapeutic target because it requires the functional neutralization of a protein that is also abundant in the circulation and tissue of healthy individuals and does not only exert proinflammatory but also physiological functions [15,26–28]. However, there is increasing evidence that it is not MIF but its oxidized isoform oxMIF that exerts the pathological functions generally attributed to MIF during inflammation. In an oxidizing inflammatory environment, MIF converts to oxMIF, which is structurally distinct from reduced MIF, and which can be exclusively found in the plasma and tissues of patients with inflammatory diseases and solid tumors [15,26,27]. OxMIF was for instance detected in the serum of SLE patients and in the urine of patients with acute LN [15]. Specifically targeting oxMIF with mAbs (BaxB01), in a rat model of GN, reduced glomerular macrophage counts, prevented crescent formation, and improved CGN symptoms [15,29]. Targeting oxMIF furthermore demonstrated a strong synergy with low dose steroids [15]. However, the first-generation anti-oxMIF mAb (imalumab) showed a short half-life in humans, increased aggregation propensity, and an unfavorable pharmacokinetic profile in a Phase 1 study in cancer patients [30]. By advanced antibody engineering *via* proprietary algorithms, we decrease the hydrophobicity and aggregation propensity of our anti-oxMIF specific antibody and as a safety measure a L234A/L235A (“LALA” mutations) was introduced in the Fc region to suppresses the binding to Fcγ receptors and complement [31].

In this study, we evaluate the therapeutic potential of the second-generation oxMIF-specific antibody ON104 in Wistar-Kyoto (WKY) rats with nephrotoxic nephritis, an established experimental model of immune-mediated CGN.

## Material and methods

### ON104 antibody

Monoclonal human anti-oxMIF antibody ON104 was produced, purified, and characterized (using a comprehensive series of assay including binding affinity, specificity, and *in vitro* safety) as described in [31,32]. Human IgG1 antibody palivizumab, purchased from THP Medical Products (PZN-10974950), was used as an isotype control IgG.

### ON104 antibody affinity evaluation

Apparent affinity of ON104 to human oxMIF and its rat ortholog was determined by direct-binding ELISA to plate-immobilized MIF (oxMIF surrogate [15]. Affinity was further assessed by Surface Plasmon Resonance (SPR) using MIF treated with ProClin300 (oxMIF surrogate) as the analyte and ON104 as the ligand. EC_50_ and K_D_ values are reported as mean ±SD according to the methods described in [32].

### *In vivo* evaluation of ON104

#### Animals

Eight to ten weeks old male Wistar Kyoto (WKY) rats (weight: 210–300 grams), from an inhouse colony at Imperial College London were used. Animals were randomized and kept under standard laboratory conditions (non-specific pathogen free) with free access to food (standard laboratory diet) and water. Automatically controlled environmental conditions were set to maintain temperature at 20–24°C with a relative humidity (RH) of 30–70%, 10–30 air changes /hr and a natural dark/light cycle (12h).

#### Ethics statement

All animal experiments were approved by local IACUC (regulations of the UK Animals, Scientific Procedures, Act 1986) and carried out under UK Home Office Project Licenses. The well-being of rats was monitored daily including normal eating, drinking, and movement. Rats were killed under isoflurane anesthesia. No additional analgesia was required.

#### Establishment of Crescentic Glomerulonephritis (CGN) and ON104 in vivo administration

Nephrotoxic nephritis (NTN) was induced in male WKY rats by single intravenous injection of 0.1 mL of nephrotoxic serum (NTS), consisting of a rabbit nephrotoxic anti-rat glomerular basement membrane (GMB) serum, obtained after subcutaneous immunization of New Zealand White rabbits with 1 mg of GMB in 1mL of complete Freund’s adjuvant (Sigma, catalog number F-5881).

**Fig 1:**
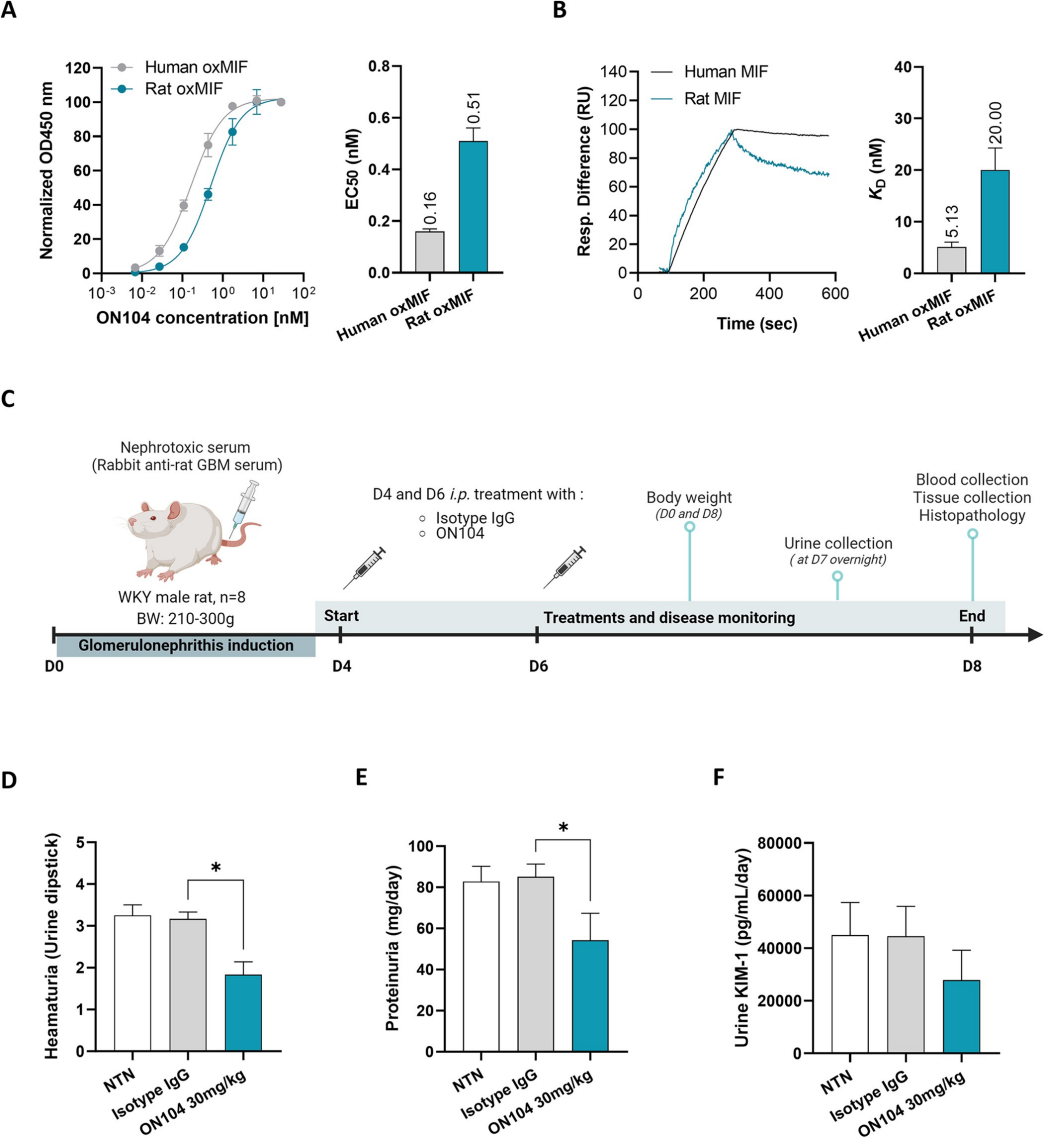
ON104 binds to rat oxMIF and ameliorates the severity of kidney injury of CGN. **(A)** ON104 antibody apparent affinity to human and mouse oxMIF (using ProClin300 to induce oxMIF structure) determined by ELISA. **(B)** ON104 affinity to human and rat oxMIF (using ProClin300 to induce oxMIF structure) by SPR. **(C)** CGN study design and treatment regimen: WKY male rats received a single injection of nephrotoxic serum (NTS, rabbit anti-rat glomerular basement membrane (GBM) serum) and at disease onset animals were treated at D4 and D6 *i*.*p*. with isotype IgG control antibody or 30 mg/kg of anti-oxMIF antibody ON104 (n = 8/treatment group). **(D)** Hematuria on day 8 post-CGN induction. **(E)** Proteinuria on day 8 post-CGN induction. **(F)** Kidney Injury Molecule (KIM)-1 levels on day 8 post-CGN in urine. Data are expressed as mean ± SEM (n = 8 animals/treatment group). Statistical analysis was performed using an ordinary one-way ANOVA test or Kruskal-Wallis test followed by Dunn’s post-test. *p < 0.05.

NTN was induced in a total of 24 rats, randomized into three groups (n = 8/group; enrolled in four staggered study cohorts): NTS control group; Isotype IgG group; and ON104 at 30mg/kg group. At disease onset (day 4), monoclonal anti-oxMIF antibody ON104 and Isotype IgG were injected intraperitoneally (*i*.*p*.), and a further dose given on day 6 after CGN induction (see Fig 1C).

At the end of treatment, overnight urine was collected using metabolic cages, sera were prepared for further kidney injury evaluation and cytokine analysis and fresh kidney tissues were harvested for subsequent histological and immune-histological analysis.

#### Evaluation of CGN severity

Severity of CGN was evaluated day 8 after disease induction using enzymatic and biochemical methods: hematuria was quantified using dipstick tests (Siemens Multistix 8 SG) by applying the following scale (1–4): 1- a positive result with ~10 erythrocytes per μL of urine; 2- a positive result with ~25 erythrocytes per μL of urine; 3- a positive result with ~80 erythrocytes per μL of urine; and 4- a positive result with ~200 erythrocytes per μL of urine. Proteinuria was measured by the sulfosalicylic acid method [33]. The presence of rat kidney injury molecule (KIM)-1, in urine, was performed by sandwich ELISA according to the manufacturer’s instructions (sensitivity about 15.6 pg/mL, DY3689, R&D Systems, USA).

#### Tissue collection and histopathology

Kidneys were fixed in 10% neutral buffered formalin, trimmed, and embedded in Paraffin prior sectioning. FFPE-sections were stained with H&E (Hematoxylin and Eosin) and PAS (Periodic acid-Schiff) for morphological evaluation. Stained tissue sections were imaged with the Leica Application Suite X Software and evaluated by light microscopy for glomerular crescent formation (20 glomeruli analyzed in each slide), and a scale of severity was used as follows: 1 –Healthy glomerulus, 2 –Less than half of the glomerulus is affected by crescent or necrosis, 3 –More than half of the glomerulus is affected by crescent or necrosis.

#### Assessment of NTS-induced antibodies in renal tissue

Deposits of the rabbit anti-rat antibody and the autologous rat IgG were detected by immunofluorescence using goat anti-rabbit IgG FITC (Sigma Aldrich, 1:100 dilution) and goat anti-rat IgG FITC (Sigma Aldrich, 1:100 dilution). Nuclei were stained blue with the addition of DAPI (Vector Labs). Slides were imaged using Leica Application Suite X software.

#### Immunohistochemistry and tissue evaluation of immune infiltrates

Immunohistochemical staining for monocytes/macrophages and neutrophils was performed in FFPE sections, after antigen retrieval. Monocytes/macrophages were stained with anti- ED-1 antibody (Biorad Mouse Anti-Rat CD68, 1:10 dilution), and detected by a HRP conjugated secondary antibody (Sigma Aldrich, ExtrAvidin Peroxidase, 1:100 dilution). Neutrophils were identified using Ly6G primary antibody (catalog no. GTX40912, GeneTex; 1:500) with a HRP secondary system. Slides were counterstained with hematoxylin. After image acquisition, using Leica Application Suite X Software, quantification of ED-1 positive cells (from a total of 20 glomeruli analyzed per section) was assessed using image analysis software (ImagePro Plus, Media Cybernetics) and expressed as a ratio between the area of positively stained cells and the total area of the glomerulus. For Ly6G staining, images at 10 or 40-fold magnification were obtained with CaseViewer 2.4 (3DHistech) and the area of positive cells per kidney cross‐section was quantified using ImageJ software and according to the method described in [34].

#### ON104 detection in renal tissue

Fresh frozen kidney tissues were OCT-embedded and cryo-sectioned prior incubation with goat anti-human HRP (catalog no. SA5-10273, Invitrogen). Images at 40-fold magnification were obtained with CaseViewer 2.4 (3DHistech,) and the area of positive cells per glomerular cross‐section was quantified using ImageJ software.

#### Human cell isolation, culture, and *in vitro* stimulation

Cells were obtained from healthy volunteers’ whole blood, purchased from the local Red Cross (Vienna, Austria). Neutrophils and monocytes were isolated by negative selection using EasySep Kit (catalog no. 19666 and 19669, STEMCELL Technologies, Canada) according to the manufacturer’s protocol. Peripheral Blood Mononuclear Cells (PBMCs) were isolated by density gradient centrifugation (SepMate catalog no. 15470, STEMCELL Technologies, Canada). Total leucocytes were obtained by adding a red blood cells lysis buffer (catalog no. 420301, Biolegend) to the whole blood (2:1; v/v) for 10 min. Isolated cells were plated in 96-well plates at a density of 0.25 x 10^6^ cells per well in RPMI medium 1640 ™ containing 5% FBS (Gibco, US), 1% PS (Gibco, US) and. 1% L-Glutamine (Gibco, USA). Cells were then incubated with or without PMA (phorbol 12-myristate 13-acetate) at a final concentration of 1 ng/mL (P8139, Sigma Aldrich, Germany) for 18 hours. Afterwards, culture supernatants were collected and stored at -80°C for further oxMIF and total MIF ELISA.

#### oxMIF/MIF-enzyme-linked immunosorbent assay (ELISA)

Total MIF (including redox neutral MIF and oxMIF) and oxidized MIF (oxMIF) levels were determined by an oxMIF-binding ELISA, according to the methods described in Ferhat *et al*., 2023; Rossmueller *et al*., 2023 with the following adaptation: ProClin300 was replaced by ProClin950 (46885-U, Sigma Aldrich, Germany); anti-oxMIF antibody described in [35], used as a capture antibody, was diluted to a concentration of 3 μg/mL in 50 mM Carbonate Buffer, 50 mM NaCl at pH 9.3 and captured oxMIF or MIF were detected with a monoclonal rabbit anti-MIF antibody (Clone A5, described in [36]) (Sysid: PT-22.0057). Absorbance (OD) was measured at 450 nm in a microplate reader (Infinite M200 PRO, Tecan). Total MIF and oxMIF levels were calculated by non-linear regression variable slope four-parameter logistic curve (4PL) in GraphPad Prism.

### Statistical analysis

All data are expressed as mean ± SEM. Following normality test, comparison between multiple groups were determined using One-way ANOVA followed by Dunnett’s post-test for parametric data (normally distributed data). For non-parametric or non-normally distributed data, Kruskal-Wallis test followed by Dunn’s post-test was performed. Comparison between two groups was determined using a Wilcoxon test. Results were considered significant at a p value lower than 0.05 (*p < 0.05; **p < 0.01; ***p < 0.001; ****p < 0.0001, and ns: not significant). Statistical tests were carried out using GraphPad Software 9.0 (GraphPad Software, San Diego, CA, USA).

## Results

### Human anti-oxMIF monoclonal antibody ON104 binds to rat oxMIF

To evaluate the binding efficiency of the ON104 antibody, directed against human oxMIF, to rat oxMIF ortholog, we determined the apparent affinity by ELISA and the affinity by SPR analysis. By ELISA ON104 binds rat oxMIF (EC_50_ = 0.51 ± 0.05 nM) with 3-times lower affinity compared to human oxMIF (EC_50_ = 0.16 ± 0.01 nM) (Fig 1A), while in solution the affinity to human oxMIF was 4-times higher indicated by *K*_D_ values of 5.13 ± 0.94 nM for human oxMIF *versus K*_D_ values of 20.00 ± 4.29 nM for rat oxMIF (Fig 1B). The different affinities might be due to sequence variations between the species in the linear core epitope of ON104 (54-**D**PCALC**-**59 for rats and 54-**E**PCALC-59 for humans).

### ON104 ameliorates severity of experimental glomerulonephritis

The therapeutic efficacy of anti-oxMIF monoclonal antibody ON104 was addressed in a robust and well-established rat model of crescentic glomerular nephritis (CGN) [37,38]. As depicted in Fig 1C, animals received a single intravenous injection of a rabbit nephrotoxic serum (NTS), targeting rat glomerular basement membrane, to induce CGN. At disease onset, rats were treated on day 4 and day 6 with ON104 at 30 mg/kg or isotype control. Animals were observed for their general health status and the treatment with ON104 was well tolerated.

On day 8, untreated and isotype control treated rats showed increased hematuria (Fig 1D; mean dipstick > 3 in both groups) as well as proteinuria (Fig 1E; over 80 mg/day), both hallmarks of kidney disease. Two *i*.*p*. treatments with ON104 were sufficient to significantly reduce hematuria, and proteinuria at the dose of 30 mg/kg (Fig 1D and 1E). Moreover, KIM-1 (kidney injury molecule-1), which is a biomarker for early and acute renal injury, was decreased in the urine of ON104 treated animals, albeit not reaching statistical significance (Fig 1F).

### ON104 prevents crescent formation and preserves glomerular morphology in CGN

**Fig 2:**
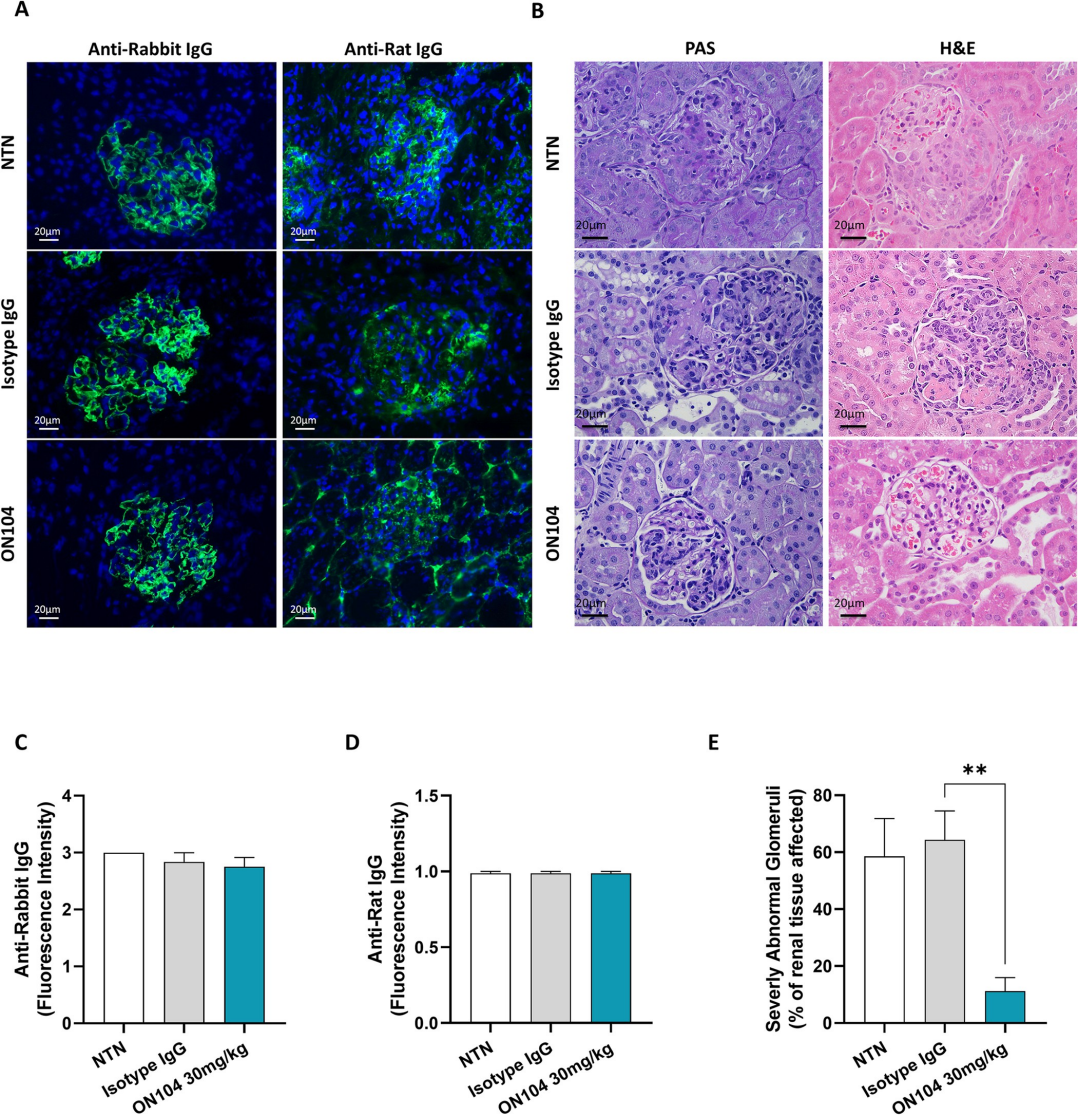
ON104 reduces crescent formation and restores normal glomeruli in CGN. Renal histology was performed on kidney tissues collected on day 8 after CGN induction **(A)** Immunofluorescent detection of anti-rabbit IgG (upper panel) anti-rat IgG (lower panel). (**B**) Representative pictures for PAS (left panel) and H&E (right panel) staining (**C-D**), fluorescent intensity of anti-rabbit IgG-stained glomeruli **(C)** and anti-rat IgG-stained glomeruli **(D)** upon NTS administration. **(E)** Percentage of severely abnormal glomeruli evaluated in 20 glomeruli/animal upon NTS administration. Pictures are at ×400 magnification and scale bar indicates 20μm. Histogram bars show n = 8 animals/treatment group and data are expressed as mean ± SEM. Statistical changes were determined using an ordinary one-way ANOVA test followed by Dunnett’s post-test or Kruskal-Wallis test followed by Dunn’s post-test. **p < 0.01.

Using direct immunofluorescence staining, we verified heterologous (rabbit) and autologous (rat) IgG deposition within glomeruli. There was no difference in fluorescence intensity within the glomeruli among all the groups for rabbit and rat IgG (Fig 2A, 2C and 2D), confirming that the disease was equally induced in all animals and that ON104 does not interfere with the humoral immune response. The main feature of renal injury during CGN is cellular crescent formation with early tubulointerstitial nephritis. Both H&E and PAS staining revealed severe histological glomerular abnormalities (Fig 2B) in kidneys from untreated (NTS) and isotype IgG-treated rats, with 60–65% of glomeruli showing global lesions (Fig 2B and 2E). Treatment with ON104 preserved glomerular morphology by restricting severe glomerular abnormalities to less than 20% in comparison to untreated and isotype IgG control where up to 60% of severely abnormal glomeruli were detected (Fig 2E).

### ON104 mitigates immune cell infiltration associated with experimental CGN

**Fig 3:**
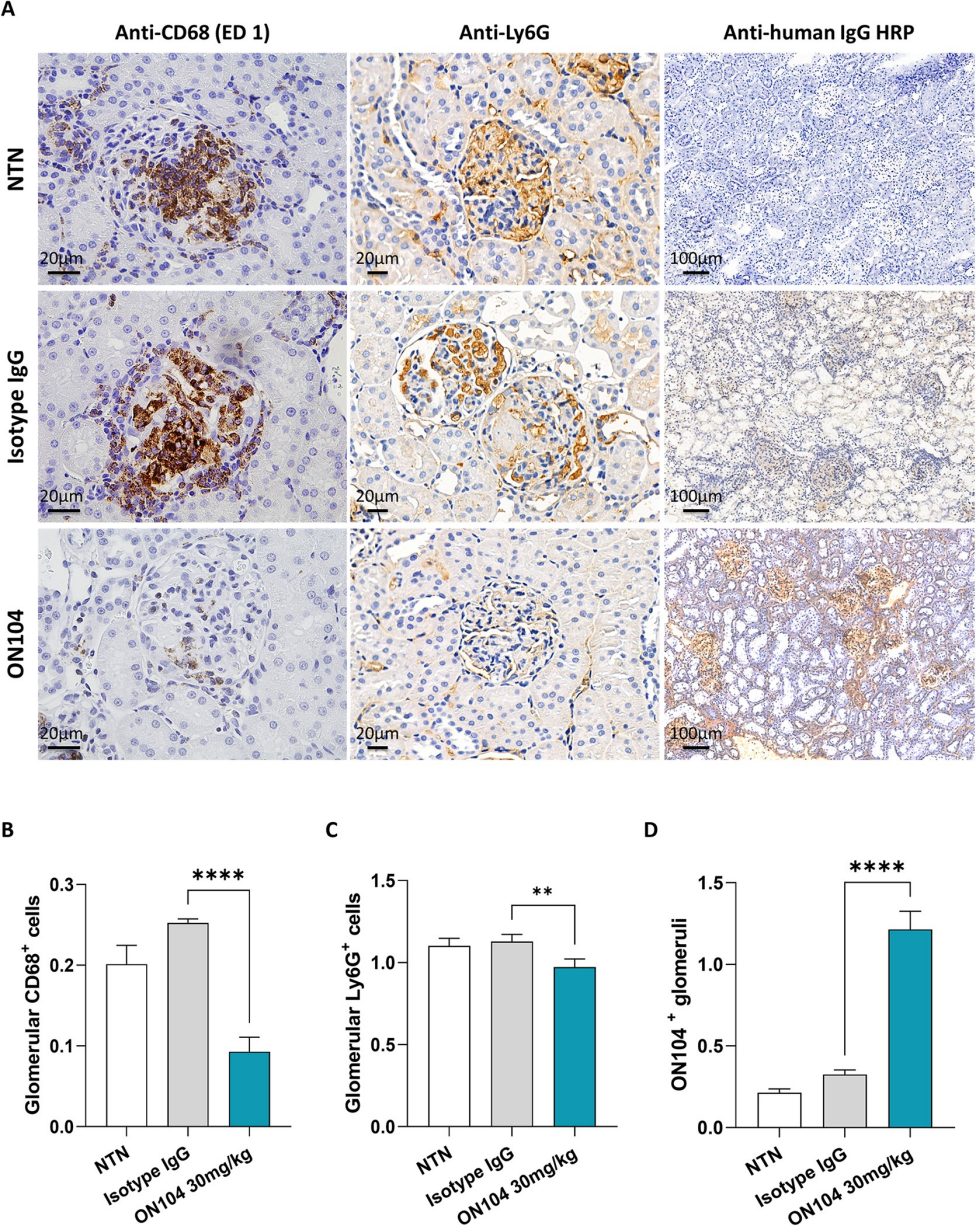
ON104 accumulates within inflamed glomeruli and reduces immune cell accumulation. Immunohistochemistry was performed on kidney tissues collected on day 8 after CGN induction. **(A)** Immunohistostaining of the kidney cortex showing CD68-positive monocyte/macrophages (left panel), Ly6G-positive neutrophils (mid- panel) (scale bar indicates 200μm), and ON104 antibody (right panel) (scale bar indicates 100μm). **(B)** CD68-positive monocyte/macrophages quantification. **(C)** Ly6G-positive neutrophil quantification; **(D)** ON104 antibody accumulation within the inflamed glomeruli. Staining intensities are in % area. Histogram bars show n = 8 animals/treatment group and express data as mean ± SEM. Statistical changes were determined using an ordinary one-way ANOVA test, followed by Dunnett’s post-test or Kruskal-Wallis test followed by Dunn’s post-test. **p < 0.01; and ****p < 0.0001.

**Fig 4:**
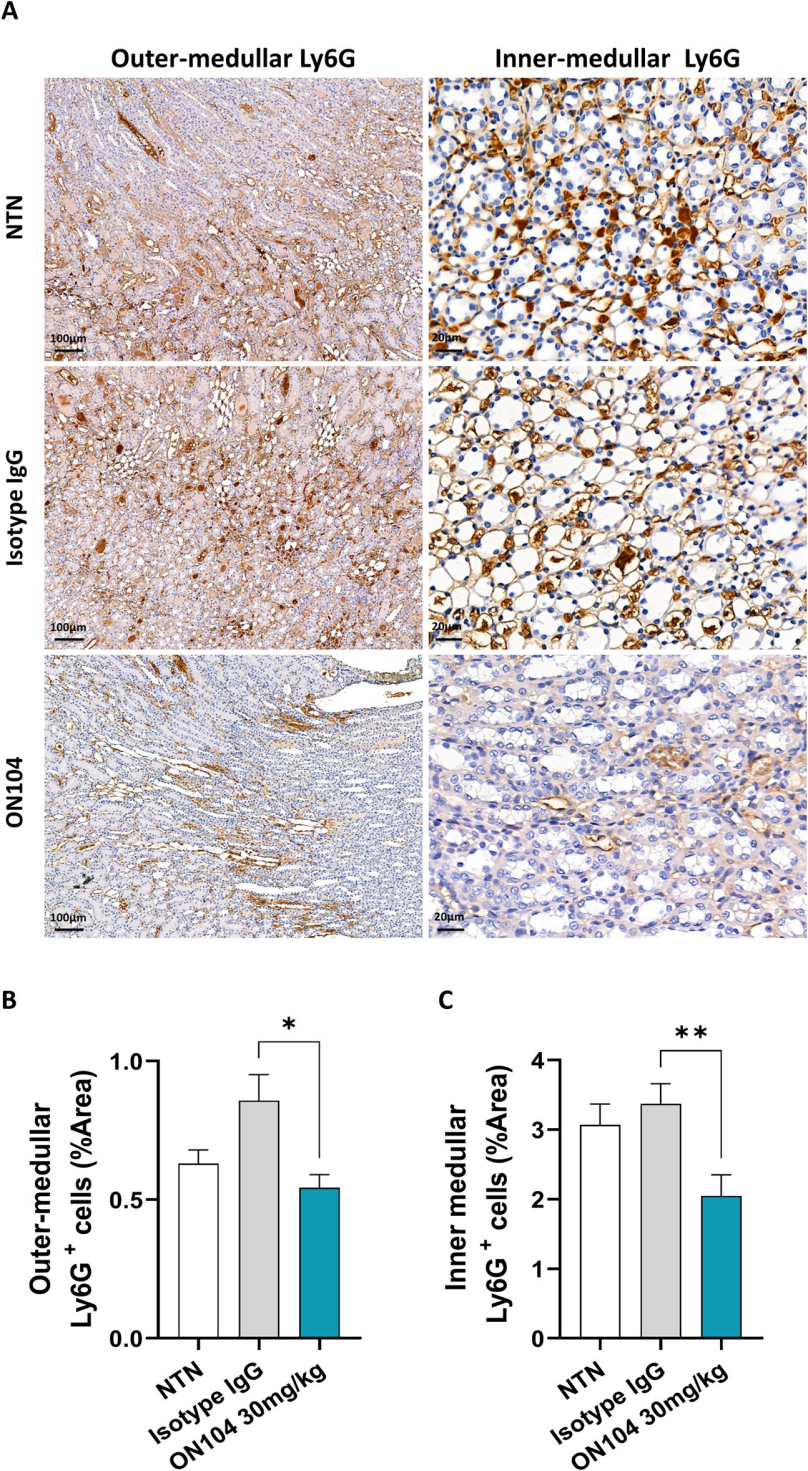
ON104 reduces Ly6G-positive neutrophil accumulation in the medulla. Neutrophil presence was assessed in the medullar area of kidney section obtained on day 8 post-CGN induction. **(A)** Immunohistostaining images for Ly6G-positive neutrophils in the outer medulla (left panel; scale bar indicates 100 μm), and inner medulla (right panel; scale bar indicates 20 μm). **(B)** Ly6G-positive neutrophil quantification in the outer medulla. **(C)** Ly6G-positive neutrophil quantification in the inner medulla. Histogram bars are from n = 8 animals/treatment group, with 20 pictures evaluated per animal (in total 160 images per group). Data are expressed as mean ± SEM. For statistical analysis a Kruskal-Wallis test followed by Dunn’s post-test was used. *p < 0.05; and **p < 0.01.

CGN is characterized by glomerular crescent formation and tissue damage injury driven by macrophage and neutrophil recruitment into the inflamed glomeruli, which ultimately leads to proteinuria and kidney failure [38,39]. By immunohistochemistry, we evaluated the effect of ON104 treatment on the number of intra-glomerular monocyte/macrophages (CD68^+^ cells) and intra-glomerular neutrophils (Ly6G^+^ cells). Administration of ON104 strongly reduced glomerular infiltration of CD68-positive monocyte/macrophages compared to isotype control-treated and untreated rats (Fig 3A top panel, and 3B). Ly6G-positive neutrophils were also significantly reduced in the glomeruli (Fig 3A middle panel, and 3C) as well as in the outer (Fig 4A left panel, and 4B) and inner medulla (Fig 4A right panel, and 4C).

### ON104 accumulates within the inflamed glomeruli

Immunostaining of the kidneys, using an anti-human IgG antibody, showed that ON104 was mainly detected in the kidney cortex. More specifically, ON104 accumulates in the glomeruli and the adjacent tubular regions (Fig 3A lower panel, and 3D), particularly in those regions where macrophages and neutrophils were detected (Fig 3A). These observations indirectly indicate that oxMIF is present at sites of inflammation where neutrophils and macrophages are present, both playing important roles in glomerular diseases.

### Neutrophils are a main source of oxMIF *in vitro*

**Fig 5:**
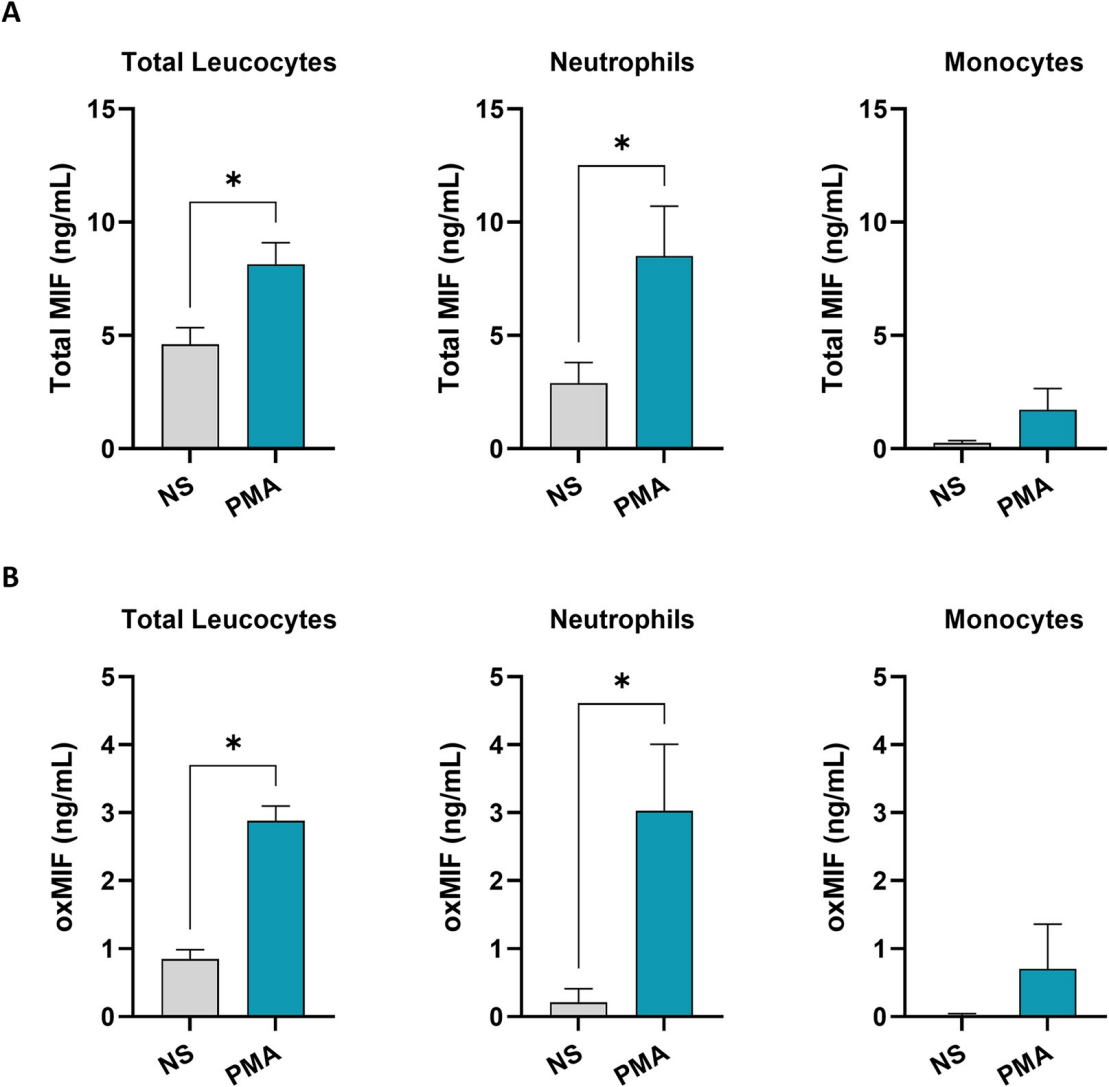
oxMIF is generated by human neutrophil upon *in vitro* stimulation. Human blood cells were freshly isolated and cultured for 18h +/- PMA (phorbol 12-myristate 13-acetate) at 1 ng/mL. **(A)** MIF levels assessed by ELISA in the cell culture supernatant of total leucocytes, neutrophils, and monocytes. **(B)** oxMIF levels assessed by ELISA in the cell culture supernatant of total leucocytes, neutrophils, and monocytes. Histogram bars represent n = 5–6 healthy donors. Data are expressed as mean ± SEM. For statistical analysis a Wilcoxon test was used. *p < 0.05.

To better understand how oxMIF is generated, total leucocytes, neutrophils, and monocytes were isolated from freshly collected human whole blood and incubated in the presence of PMA for 18 hours. Under non-stimulating (NS) conditions, a constitutive secretion of total MIF was detected in the cell culture supernatant of total leucocytes (4.6 ng/mL ± 0.73), isolated neutrophils (2.89 ng/mL ± 0.91) and lower levels in supernatants from isolated monocytes (0.24 ng/mL ± 0. 11) (Fig 5A). Stimulation with PMA induces approximately a 2-fold increase of total MIF levels in leucocytes and a 3-fold increase in neutrophils compared to the baseline (Fig 5A). Interestingly, the increase of oxMIF levels was more pronounced, in total leucocytes 2.88 ng/mL ± 0.21 *versus* NS at 0.84 ng/mL ± 0.13, corresponding to a 4-fold increase, and in neutrophils a 14-fold rise from 0.21 ng/mL ± 0.19 in NS *versus* 3.02 ng/mL ± 0.98 was seen (Fig 5B). In general, in monocytes the stimulation with PMA triggered a lower increase of MIF and oxMIF levels (Fig 5A and 5B). These data support the hypothesis that among peripheral human immune cells neutrophils are a prominent source of oxMIF generation under inflammatory conditions.

## Discussion

The present study demonstrates the immunosuppressive activity of the anti-oxMIF antibody ON104 in a rat model of CGN, thereby preventing severe kidney damage and loss of renal function by reducing immune cell infiltration. This protective effect of ON104 was associated with reduced numbers of glomerular monocytes/macrophages as well as neutrophils, despite the glomerular deposition of humoral immune complexes and a pre-existing systemic immune response to rabbit IgG. ON104 treatment also significantly reduced neutrophil infiltration in both the renal cortex (the glomerular-enriched area) and the medullar area. Since inflammation in the extra-glomerular tissue is increasingly recognized to be of importance in the pathogenesis of CGN this is likely to be a significant therapeutic effect of ON104. Altogether, our data demonstrate that neutralizing oxMIF alleviates renal inflammation *in vivo* and reinforces earlier observations from a first-generation anti-oxMIF antibody (BaxB01) [15,29] In contrast to the first-generation anti-oxMIF antibody, our second-generation anti-oxMIF antibody ON104 was engineered to be less hydrophobic with a lower propensity to aggregate, leading to increased bioavailability and half-life [31]. Compared to the first-generation anti-oxMIF antibody, “LALA" mutations (L234A/L235A) were introduced into the Fc region to minimize binding to Fcγ receptors and complement. Consequently, ON104 is a highly specific oxMIF-neutralizing antibody devoid of effector functions, as previously demonstrated by the lack of antibody-dependent cell-mediated cytotoxicity (ADCC) and complement-dependent cytotoxicity (CDC) [31]. The present study shows for the first time the efficacy of the second-generation oxMIF-specific antibody ON104 in a rat model of CGN and substantiates that oxMIF is a highly attractive therapeutic target in glomerulonephritis.

Historically, MIF was identified as a T-cell product, but nowadays various immune cells have been proven to increase their MIF content upon activation [40]. However, the distinction between MIF and its oxidized isoform oxMIF has not yet been addressed. Here, we demonstrate for the first time that immune cells, and in particular neutrophils produce oxMIF during PMA-induced oxidative stress and inflammation. This is in line with previous observations showing increased oxMIF presence on the cell surface of neutrophils from patients with sepsis compared to healthy subjects where oxMIF was absent [15]. In addition, *in vitro* oxidized MIF by hypochlorous acid, was able to prolong neutrophil survival *in vitro* [41]. Conversely, neutralization of oxMIF by ON104 may have shortened the survival time of neutrophils and thus contributed to the depletion of neutrophils in the inflamed tissue providing a further potential mechanism of action of ON104 in decreasing the severity of CGN. With their detrimental release of inflammatory substances, such as MPO, ROS, cytokines, and neutrophil extracellular traps (NETs), neutrophils are pivotal players in CGN and other autoimmune diseases [42]. However, we cannot exclude the role of intrinsic kidney cells, such as glomerular mesangial cells, and tubular epithelial cells as well as other resident-immune cells that might also be an important source of oxMIF or contribute to oxMIF *in situ* generation.

In patients with SLE, circulating levels of oxMIF and total MIF, were found to be elevated in patients with disease flare compared to patients in remission [15]. A correlation between oxMIF levels and disease severity and treatment outcomes in patients with glomerular diseases could become a helpful tool for patient stratification and application of the appropriate medication in the future but is currently unexplored.

Conventional treatment strategies for glomerular diseases involve high-dose and long-term administration of glucocorticoids (GCs) (e.g. prednisone). and cytotoxic medications (e.g. cyclophosphamide), despite their significant adverse effects and potentially deleterious effects in patients with maladaptive focal glomerulosclerosis [43]. When inappropriately dosed, GC treatment can result in steroid resistance, which remains a severe medical challenge and frequently involves life-threatening conditions. MIF has been shown to modulate GC responses in several clinical and pre-clinical studies. In patients with SLE, a positive correlation between circulating MIF levels and GCs resistance has been reported [20]. Moreover, variability in glomerular expression patterns of GC receptors among patients might influence GC responsiveness/resistance [44,45]. In this context, MIF is well recognized to counteract the immunosuppressive effects of GCs *via* altering nuclear factor-κB (NFκB) transcriptional activity and by overriding the glucocorticoid-mediated inhibition of PLA2 activity and arachidonic acid production [40,46,47]. Therefore, resistance to GC therapy may be explained by the regulatory effects of MIF on downstream effector mechanisms of GCs. *In vitro* studies demonstrated that anti-oxMIF antibodies inhibit the GC-overriding activity of (ox)MIF [48]. In line with this data, a synergistic effect of anti-oxMIF antibodies and sub-therapeutic doses of dexamethasone was previously demonstrated in CGN rats [15]. Based on these findings, we anticipate that ON104 has the potential to become a GC-sparing therapy for GN patients. This would open new perspectives for glomerular disease management, as it would enable lower dosing of standard of care, thus enhancing therapy and/or overcoming steroid resistance.

Herein we show that oxMIF plays a substantial role in CGN and that its pathological functions can be successfully inhibited with ON104. Notably, ON104 leads to a substantial reduction in the population of active macrophages/monocytes, consistent with previous findings in a collagen-induced arthritis model [31], and a decrease in neutrophil infiltration within the inflamed renal tissue. Additionally, we have established evidence that among immune cells, neutrophils are a predominant source of oxMIF. These data underscore that neutralization of oxMIF by ON104 could be a new therapeutic option for glomerulonephritis, particularly those in which neutrophils and tissue macrophages play crucial, disease-promoting roles.
